# Younger women and stroke: why person-centred rehabilitation must be more than a concept

**DOI:** 10.1177/17449871251370621

**Published:** 2025-09-17

**Authors:** Pernilla Garmy

**Affiliations:** Professor, RN, Kristianstad University, Sweden

Commentary on: Tailored Stroke Rehabilitation for Younger Women: The MENTOR HUB Framework (Tarihoran et al., 2025)

Although stroke is often associated with older adults, younger individuals can also be affected. The present study (Tarihoran et al., 2025) highlights a particularly vulnerable group – young women who experience stroke – and aims to develop a new rehabilitation framework tailored specifically to their needs.

Stroke refers to conditions caused by a blood clot or bleeding in the brain, leading to oxygen deprivation and sudden loss of functions such as speech, motor control, sensation, and vision. Research shows that women not only have a higher lifetime risk of stroke than men but also tend to live with more significant post-stroke disability and poorer long-term outcomes (Fleming et al., 2025). These gender-specific disparities must be addressed in the development of equitable rehabilitation strategies.

The study (Tarihoran et al., 2025), conducted in New Zealand, is based on qualitative data from five female stroke survivors aged 18–64 and five healthcare professionals, including nurses and occupational therapists. Using a two-stage approach, focus groups and interviews were first conducted, followed by a co-creative process to design the rehabilitation framework.

The result is the MENTOR HUB Framework, where MENTOR stands for:

- Monitoring – monitoring of risk factors
- Education – education on stroke, prevention, and women’s health
- Navigation – guidance in navigating a complex healthcare system
- Tools – resources for self-management and rehabilitation
- Ongoing Support – continuous assistance and guidance
- Recovery programme – a holistic recovery plan addressing physical, psychological, social, cultural, and spiritual dimensions

HUB stands for *Holistic*, *User-centred* and *Be-continuous*, underscoring the need for care that is integrated, tailored and sustained over time.

## Reflection and links to practice and theory

I read this study with both professional and personal engagement. As the daughter of a stroke patient – my mother – I witnessed first-hand the crucial role of rehabilitation, and the importance of tailoring care to the individual. Observing a loved one lose basic functions is deeply distressing, and recovery requires not only time and medical care but also human presence, emotional support, and continuity.

In my professional roles – both in paediatric hospital care and as a school nurse – I have also encountered children who have had strokes. Although rare, it does occur. These experiences have taught me that every patient must be seen in their unique context. This is especially true for younger women with stroke, whose needs often differ from both men and older patients.

Several studies indicate that women have worse long-term outcomes after stroke compared to men, including a higher degree of disability and an increased risk of mental health problems such as depression (Fleming et al., 2025). This, in turn, may impair their ability to engage fully in rehabilitation, creating a vicious cycle. As current care models are typically designed around an older, gender-neutral patient profile, younger women may be inadvertently overlooked.

The MENTOR HUB framework closely aligns with principles of person-centred nursing, where the patient’s experiences, values and goals are at the heart of care planning and delivery. McCance and McCormack (2025) emphasised that person-centred care is not only about recognising individuality but also about fostering workplace cultures that support relational and context-aware practices. The MENTOR HUB framework operationalises many of these principles – particularly continuity, participation, and holistic recovery – offering a practical and scalable model for stroke rehabilitation grounded in nursing values.

## Practical implications: Who will benefit and how?

The MENTOR HUB framework is a timely and valuable addition to stroke care, with relevance for several stakeholder groups. First and foremost: the patients themselves. The framework is informed by the lived experiences of younger female stroke survivors, representing a clear embodiment of person-centredness.

Healthcare professionals – especially nurses, physiotherapists, occupational therapists, and social workers – can use the framework as a concrete tool in care planning, communication, and follow-up. In educational settings, the framework offers a useful lens for examining gender- and age-specific needs within stroke rehabilitation.

Finally, policymakers and healthcare leaders should take note of this research. The implementation of MENTOR HUB into clinical guidelines and service planning could help reduce disparities in stroke outcomes and improve quality of life for a group that has long been underrepresented in research and practice.

I sincerely hope this framework gains traction in clinical settings and resources are allocated to support its adoption. Women of working age who experience stroke deserve a rehabilitation process that reflects their reality.
